# Comments on “A New Elliptical Model for Device-Free Localization”

**DOI:** 10.3390/s18030715

**Published:** 2018-02-27

**Authors:** Yanan Yuan, Wei Ke, Jie Jin, Jun Lu, Jianhua Shao

**Affiliations:** 1 Jiangsu Key Laboratory on Opto-Electronic Technology, School of Physics Science and Technology, Nanjing Normal University, Nanjing 210023, China; 151035011@stu.njnu.edu.cn (Y.Y.); 161035013@stu.njnu.edu.cn (J.J.); 161002040@stu.njnu.edu.cn (J.L.); shaojianhua@njnu.edu.cn (J.S.); 2 Jiangsu Center for Collaborative Innovation in Geographical Information Resource Development and Application, Nanjing 210023, China

**Keywords:** elliptical model, device-free localization, fresnel diffraction theory

## Abstract

A recent paper “A New Elliptical Model for Device-Free Localization” (Sensors 2016, 16, 577) presents a new geometry-based elliptical model for Device-free localization (DFL). In this comment, we point out some problems of the original paper and exploit the same data used in the original paper to demonstrate the existence of these problems. Then, we give a modified formula to correct their model. Meanwhile, a real experiment is performed to verify our conclusion.

## 1. Introduction

In [1], the authors propose a new geometry-based elliptical model to improve the accuracy of device-free localization (DFL). Different from the traditional elliptical model in [2], weights are different in the different areas in their model. Moreover, the authors expressed that, when a target stands on a line-of-sight (LOS) path, the influence on the communication link is greater than when a person stands on a non-line-of-sight (NLOS) path inside the same weighting area. The essence of this model is to construct the following formula ([1] Equation (10)).

(1) $$W_{ij}^{k}=\{\begin{array}{cc} \frac{1}{d}(k_{1}+\max(d_{ij}^{k}(1),d_{ij}^{k}(2)), & \mathrm{if}\;d_{ij}^{k}(1)+d_{ij}^{k}(2)<d+\lambda ,\;d_{ij}^{k}(1)+d_{ij}^{k}(2)\neq d\\ \frac{1}{d}(k_{2}+\max(d_{ij}^{k}(1),d_{ij}^{k}(2)), & \mathrm{if}\;d_{ij}^{k}(1)+d_{ij}^{k}(2)<d+\lambda ,\;d_{ij}^{k}(1)+d_{ij}^{k}(2)=d\;\\ 0, & otherwise \end{array}$$

where $W_{ij}^{k}$ is the weight of voxel V*_ij_* in link *k*.$k_{1}$ is a coefficient representing the obstacle to communication on the non-line-of-sight path, whose value is 2 by empirical experiments. $k_{2}$ is a coefficient representing the obstacle to communication on the line-of-sight path, whose value is 2.5 by empirical experiments. $d_{ij}^{k}(1)$ and $d_{ij}^{k}(2)$ are the distance of the voxels and the two nodes. d is the distance of the two nodes. The correct weight model is the basis of DFL. However, this formula is contrary to the description in their paper [1] that, when a person stands on a LOS path, the influence on the communication link is greater than when a person stands on a NLOS path inside the same weighting area.

## 2. Analysis of the Problems in the Original Paper

**Figure 1:**
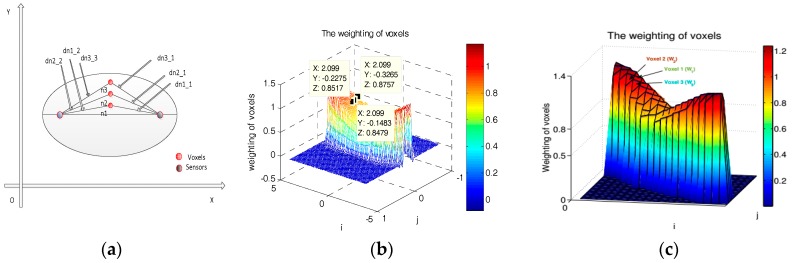
(**a**) An example of three voxels in the ellipse; (**b**) the simulated shadowing effect map by Formula (1); and (**c**) the authors’ result for Formula (1) (i.e., Figure 3c in [1]).

In Figure 1a, n1, n2, and n3 are three voxels in the ellipse that they have the same abscissa. It is obvious that the weight of voxel n3 is biggest among the three voxels according to Formula (1). Furthermore, we give the weight distribution of a link in Figure 1b, which also shows the weight of the voxel that near the LOS path is smaller than those voxels that are far from the LOS path. These phenomena show Formula (1) is conflicting to the authors’ purpose. Interestingly, Formula (1) is also contradictory to the authors’ result in [1]. As shown in Figure 1c (i.e., Figure 3c in [1]), it is obvious that the weight increases from the elliptical edge to LOS path of the ellipse. However, the trend of the weight in Formula (1) is not consistent with Figure 1c.

**Figure 2:**
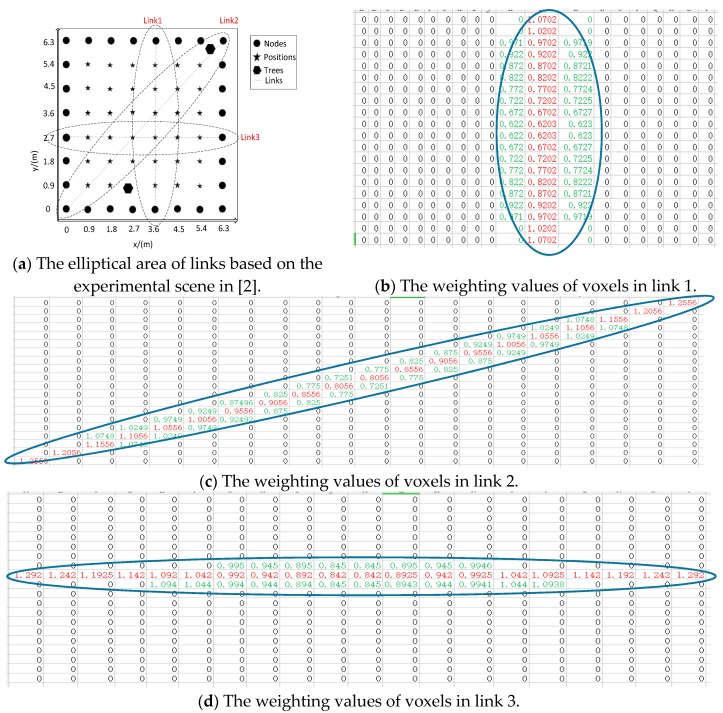
Examples of Formula (1): the weighting values of voxels in the LOS path are marked in red font and the weighting values of voxels in the non-line-of-sight (NLOS) path are marked in green font.

The more serious problem of their model is that the proposed Formula (1) (i.e., Equation (10) in [1]) is not always consistent with their description “*when a person stands on a line-of-sight path, the influence on the communication link is greater than when a person stands on a non-line-of sight path inside the same weighting area*” in their paper [1]. To verify this, we use the same parameters k_1_ and k_2_ in [1] to calculate the weighting values of voxels according to Formula (1), as shown in Figure 2.

In Figure 2, it can be clearly found that only the weighting values of link 2 are fit for the above description. However, in link 1 and link 3, the weighting values of voxels in the LOS path is less than the weighting values of voxels in the NLOS path (although the differences are small), which is contrary to the description “*when a person stands on a line-of-sight path, the influence on the communication link is greater than when a person stands on a non-line-of sight path inside the same weighting area*”.

The authors emphasized that their proposed elliptical model in [1] has been developed based on the work in [2], and they used the same experiment scenario and data as in [2] to design the new elliptical model. However, after we read Papers [1] and [2] carefully, we find that, in [2], the experiment is conducted by a sensor network consisting of 28 sensors which is deployed in a grassy area along the perimeter of a 21 × 21 foot square (i.e., about 6.3 m × 6.3 m), while, in [1], the authors describe “The monitoring area was 7 m × 7 m, …, which is shown in Figure 4a”. This is obviously inconsistent with their description about using the same experiment scenario as [2]. We advise the authors to prove the effectiveness of their results in the right experimental area (i.e., 6.3 m × 6.3 m), because the coordinate errors between “6.3 m × 6.3 m (about 39.7 m^2^)” and “7 m × 7 m (49 m^2^)” are too large for small-area DFL.

## 3. Correction

In fact, based on the well-known Fresnel diffraction theory [3], about 90% of the propagation energy between the transmitting and receiving nodes is within the first Fresnel zone of the link, which distributes around the LOS path. According to this theory, the distance between a target and a LOS path is smaller, and the influence of the target is greater. Therefore, Formula (1) is incorrect and should be replaced by

(2) $$W_{ij}^{k}=\{\begin{array}{cc} \frac{1}{d}(k_{1}+1/\min(d_{ij}^{k}(1),d_{ij}^{k}(2)), & \mathrm{if}\;d_{ij}^{k}(1)+d_{ij}^{k}(2)<d+\lambda ,\;d_{ij}^{k}(1)+d_{ij}^{k}(2)\neq d\\ \frac{1}{d}(k_{2}+1/\min(d_{ij}^{k}(1),d_{ij}^{k}(2)), & \mathrm{if}\;d_{ij}^{k}(1)+d_{ij}^{k}(2)<d+\lambda ,\;d_{ij}^{k}(1)+d_{ij}^{k}(2)=d\;\\ 0, & otherwise \end{array}$$

**Figure 3:**
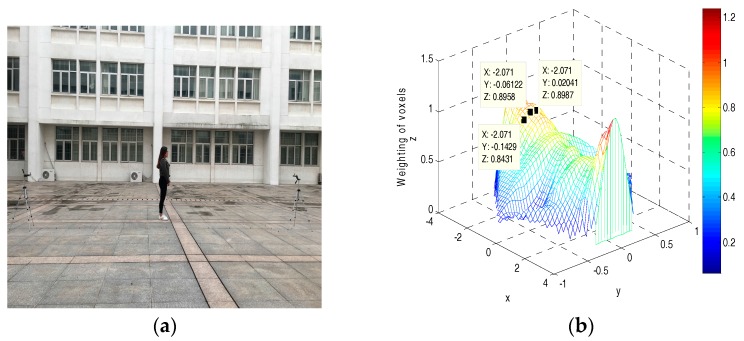
(**a**) Experimental setup; and (**b**) the measured shadowing effect map.

To further verify our conclusion, a real experiment is performed to validate the proposed new model. The experimental system is made up of the two wireless nodes shown in Figure 3a, and they operate on a 2.4-GHz Industrial Scientific Medical (ISM) band. From the visualized results in Figure 3b, we can see that the obtained shadowing effect map is consistent with the new elliptical model.

Finally, we must emphasize that the object of our comment is to correct the original paper’s problems rather than propose a new model. Therefore, Formula (2) is also not a perfect model, which only overcomes some shortcomings of Formula (1).

## References

[1] Qian L., Haijian Z. (2016). A New Elliptical Model for Device-Free Localization. Sensors.

[2] Patwari N., Wilson J. (2010). RF Sensor Networks for Device-Free Localization: Measurements, Models, and Algorithms. IEEE Proc..

[3] Rappaport T.S. (2009). Wireless Communications: Principles and Practice.

